# Research on the influence of patient cost-sharing on medical expenses and health outcomes: Taking patients with heart failure as an example

**DOI:** 10.3389/fpubh.2023.1121772

**Published:** 2023-03-14

**Authors:** Huyang Zhang, Ke Ning, Jinxi Wang, Hai Fang

**Affiliations:** ^1^Department of Health Policy and Management, Peking University School of Public Health, Beijing, China; ^2^China Center for Health Development Studies, Peking University, Beijing, China; ^3^Institute for Global Health and Development, Peking University, Beijing, China; ^4^China Center for Health Economic Research, Peking University, Beijing, China; ^5^School of Public Health, Li Ka Shing (LKS) Faculty of Medicine, The University of Hong Kong, Hong Kong, Hong Kong SAR, China; ^6^Shanghai Songsheng Business Consulting Co. Ltd, Beijing, China; ^7^Peking University Health Science Center-Chinese Center for Disease Control and Prevention Joint Center for Vaccine Economics, Peking University, Beijing, China

**Keywords:** heart failure, health insurance reimbursement policies, Urban Employees' Basic Medical Insurance, medical expenses, health outcomes, cost-sharing ratios

## Abstract

**Objective:**

The objective of this study is to assess the impact of the changes in patient cost-sharing on the medical expenses and health outcomes of patients with heart failure in China.

**Methods:**

The claim data of patients diagnosed with heart failure enrolled in the Urban Employees' Basic Medical Insurance (UEBMI) in the Zhejiang province, China, was used, covering the period from 1 January 2013 to 31 December 2017. The impact of the policy change was estimated through the use of the difference-in-differences method and the event study method.

**Results:**

A total of 6,766 patients and their electronic health insurance claim data were included in the baseline year of 2013. Following the change in the UEBMI reimbursement policies (policy change), a notable decrease was observed in the patient cost-sharing ratios, particularly in the copayment ratio within the policy. However, it did not result in a reduction of the out-of-pocket ratio, which remains a primary concern among patients. An increase was observed in annual outpatient medical expenses, while annual inpatient medical expenses decreased, leading to higher annual medical expenses in the treatment group in comparison to the control group. The effect of the UEBMI reimbursement policy change on health outcomes showed a reduction in the rehospitalization rate within 90 days; however, no significant impact was seen on the rehospitalization rate within 30 days.

**Conclusion:**

The impact of the policy change on medical expenses and health outcomes was found to be modest. To effectively address the financial burden on patients, it is crucial for policymakers to adopt a comprehensive approach that considers all aspects of medical insurance policies, including reimbursement policies.

## 1. Introduction

Research into the impact of patient cost-sharing on patient medical expenses and health outcomes has been a subject of significant interest within the field of health economics. One of the most seminal studies in this area is the RAND Health Insurance Experiment (RAND HIE), which was initiated in the United States in 1971 (1). The RAND HIE demonstrated that medical care utilization increased with a reduction in patient cost-sharing, as evidenced by an elasticity of −0.2 (2–4). This conclusion has been supported by numerous subsequent studies—with different elasticity sizes—including the Oregon Health Insurance Experiment (Oregon HIE), which was initiated in the United States in 2008 (5–15). However, the evidence is not consistent regarding the impact of patient cost-sharing on health outcomes. Some studies, such as the RAND HIE and the Oregon HIE, have shown no significant impact on participants' health (5, 8, 10), while others have reported lower mortality rates or improved health among those with lower cost-sharing (12, 16). In China, Huang and Gan found a significant decrease in both outpatient service utilization and medical expenditure but no significant effect on self-assessed health, following the implementation of a policy change in 1998, which involved an increase in patient cost-sharing through the transition from the previous labor insurance medical system to the new urban employees' medical insurance system (13). These inconsistencies may be attributed to heterogeneity in the demand response to medical services across different population subgroups, such as those with various diseases or health statuses. The current body of literature primarily focuses on evaluating the impact of patient cost-sharing on medical utilization or expenses, while there is a limited number of studies examining its effect on health outcomes and focusing on a specific disease (11, 15).

The American Economic Review paper in 2022 discovered that various diseases displayed varying patterns, where some, like heart failure, had a positive productivity growth, whereas others, such as musculoskeletal conditions, did not (17). This highlights the need for a more tailored approach to guide policymaking, as a one-size-fits-all approach may not be effective in addressing the needs of all patient populations. Policymakers need to consider the unique characteristics of different diseases to achieve the maximum effect of policy decisions. The prevalence of heart failure, an Ambulatory Care Sensitive Condition (ACSC), has been increasing globally, with 4.49 per 1,000 persons in 2019 according to the Institute for Health Metrics and Evaluation (iHME) data. This reveals a progressively larger population with heart failure, especially among older adults (18). With the population of China surpassing 1.4 billion and urging an aging problem, medical care utilization among heart failure patients is unignorable. Congestive heart failure, as the main type of heart failure, imposes the greatest direct and indirect financial burden among 30 major diseases, accounting for 9.96% of China's national health costs in 2008 and also ranked among the top 10 in 2013 (19, 20). Chernew et al. (21) conducted a study using data from the United States and found that higher cost-sharing for heart failure patients resulted in decreased medication use and subsequently lower medical expenses, particularly in low-income areas. In a separate study conducted in the United States, Snider et al. (22) found that the increase in cost-sharing was associated with greater per-patient cost increases for individuals with both diabetes and heart failure compared to those with diabetes alone. However, research on how patient cost-sharing affects medical expenses or health outcomes in heart failure populations is limited.

Therefore, this study aims to contribute to the existing evidence concerning the impact of changes in patient cost-sharing on the medical expenses and health outcomes of patients with heart failure using health insurance claims data in China. To our knowledge, this is one of few studies on the impact of patient cost-sharing in a specific disease population in a developing country using large provincial representative datasets at the patient level. From a public health perspective, this study provides critical knowledge for all stakeholders to better understand the impact of insurance reimbursement policies among patients with heart failure in China. Specifically, for policymakers, this knowledge is critical to informing potential policy shifts to reduce the disease burden.

## 2. Methods

### 2.1. Policy introduction

This study uses a natural experiment of policy change in some cities of the Zhejiang province, China to study the impact of patient cost-sharing on medical expenses and health outcomes among patients with heart failure. The health insurance reimbursement policies of five cities in the Zhejiang province underwent changes between the years 2013 and 2017, with the aim of alleviating the financial burden on patients. These changes included increasing the reimbursement rate, raising the cap line, and decreasing the deductible line of the Urban Employees' Basic Medical Insurance (UEBMI). The summary of these modifications is illustrated in Table 1. As the policy change in Shaoxing city took place in 2017, which was late compared to the other four cities, Shaoxing city was treated as a control group after excluding their data in 2017. In addition, Lishui city was omitted due to a lack of data.

**Table 1:** Changes in Urban Employees' Basic Medical Insurance reimbursement policies between 2013 and 2017 in prefecture-level cities in the Zhejiang province.

**Prefecture**	**Ever change**	**Date of new policy**	**What changed**	**Government document**
Hangzhou	YES	Jan 1, 2014	Change A and Change B	No.68 (2013) (23) No.8 (2013) (24)
Ningbo	NO	–		–
Wenzhou	NO	–		–
Jiaxing	YES	Jan 1, 2015	Change A and Change B	No.87 (2014) (25)
Huzhou	NO	–		–
Shaoxing	YES	Jan 1, 2017	Change A	
Jinhua	YES	Jul 1, 2014	Change A and Change B	No.40 (2014) (26)
Quzhou	NO	–		–
Zhoushan	NO	–		–
Taizhou	YES	Aug 1, 2015	Change A, Change B, and Change C	No.17 (2015) (27)
Lishui	NO	–		–

Shaoxing city was selected as the control group after excluding its data from the year 2017; Lishui city was omitted due to a lack of data. Change A refers to “increasing the reimbursement rate”, Change B refers to “raising the cap line”, and Change C refers to “decreasing the deductible line”.

### 2.2. Data source and study population

The data used in this study was obtained from the UEBMI Database, which had undergone the deidentification of individual information. The database was established and managed by the Chinese Ministry of Human Resources and Social Security, and localities regularly reported to the ministry on a monthly basis. After the national institutional reform in 2018, the ministry-related responsibility was transferred to the newly established National Medical Security Bureau (28).

For the purpose of this study, patients diagnosed with heart failure through either inpatient or outpatient visits were included, using the ICD-10 code I50 and its corresponding Chinese name in medical insurance records as criteria. Individuals younger than 20 years old were excluded. A stratified sampling method was applied to a sample of all heart failure patients' data. The primary stratum was prefecture-level cities in the Zhejiang province. Within each prefecture-level city, the population was stratified by gender and age. Simple random sampling of 1% was conducted within each stratum. The data were aggregated into annual panel data to form the sample for this study.

### 2.3. Outcome measures

In this study, three types of dependent variables were used: (1) Medical expenses, (2) Cost-sharing ratios, and (3) Health outcomes (rehospitalization rate). The medical expenses comprised the total expenses, which were further divided into self-expenses within policy, insurance expenses within policy, and self-expenses not covered by policy. The out-of-pocket expenses were calculated as the sum of self-expenses within policy and self-expenses not covered by policy. The cost-sharing ratios included the ratio of self-expenses within policy on total expenses, the out-of-pocket ratio, and the copayment ratio within policy. The health outcomes were measured as the rehospitalization rate within 30 and 90 days, which are widely used indicators (29–32). The definition of each index is presented in Table 2. Patients' sex, age, and comorbidities were included as control variables.

**Table 2:** Definition of outcome variables.

**Variable**	**Number**	**Calculation**
**Type1: medical expenses**		
Total expenses	a	= b + c + d
Self-expenses within policy	b	
Insurance expenses within policy	c	
Self-expenses not covered by policy	d	
Out-of-pocket expenses	e	= a – c = b + d
**Type2: cost-sharing ratios**		
Ratio of self-expenses within policy on total expenses	f	= b/a
Out-of-pocket ratio	g	= e/a
Copayment ratio within policy	h	= b/(b + c)
**Type3: health outcomes**		
Hospitalization rate		
Rehospitalization rate within 30 days		
Rehospitalization rate within 90 days		

“Policy” means the reimbursement policies of Urban Employees' Basic Medical Insurance.

### 2.4. Study design and statistical models

The difference-in-differences (DID) method is a widely used method of analysis for evaluating the impact of exogenous shocks such as policy changes. This method was first introduced by Ashenfelter (33) as a way to assess the impact of education and training programs on income. It divides the sample into two groups: a treatment group that is subject to the shock (after the shock occurs) and a control group that is not. The DID method assumes that if the treatment group were not subject to the shock, it would have had similar trends in variables as the control group.

In this study, the DID method was applied to two types of data. The first specific regression was the main model, which used the annual panel data. The model was as follows:


(1) $$\begin{array}{l} Y_{iy}=\beta_{0}+\beta_{1}Treated_{iy}+\beta_{2}Postpolicy_{iy}+\beta_{3}Postpolicy_{iy}\\ \;{}^{*}Treated_{iy}+\beta_{4}Control_{iy}+\varepsilon_{iy} \end{array}$$

In this model, i in *Y*_*iy*_ denoted patient ID and y denoted year, forming a patient-year panel data. *Y*_*iy*_ was the dependent variable, which represented various medical expenses, cost-sharing ratios, and health outcomes, as shown in Table 2. To account for the skewed distribution of medical expenses, all indices of medical expenses were transformed by the natural logarithm (34). *Treated*_*iy*_ was a dummy variable that took a value of 1 if the patient was in the treatment group (i.e., they were from one of the four cities where the health insurance reimbursement policies changed) and a value of 0 if they were in the control group (i.e., they were from one of the other six cities where the health insurance reimbursement policies did not change). *Postpolicy*_*iy*_ represented the change in health insurance reimbursement policies and took a value of 0 if the change had not occurred yet, 1 if the whole year was after the policy change, or (12-M + 1)/12 if the change started in the Mth month of the current year. The interaction term $Postpolic{y_{iy}}^{*}Treated_{iy}$was of interest as it indicated whether the change in health insurance reimbursement policies in the treatment group had an impact on *Y*_*iy*_ after considering the time effect of the control group and, if so, the direction of the impact. *Control*_*iy*_ indicated a set of control variables such as age, gender, and 31 dummy variables for various comorbidities, which were described by Quan et al. (35) and can be found in Table 3. ε_*iy*_ was the error term.

**Table 3:** Elixhauser disease categories.

**Number**	**Disease name**
1	AIDS/HIV
2	Alcohol abuse
3	Blood loss anemia
4	Cardiac arrhythmias
5	Chronic pulmonary disease
6	Coagulopathy
7	Congestive heart failure
8	Deficiency anemia
9	Depression
10	Diabetes, complicated
11	Diabetes, uncomplicated
12	Drug abuse
13	Fluid and electrolyte disorders
14	Hypertension, complicated
15	Hypertension, uncomplicated
16	Weight loss
17	Hypothyroidism
18	Liver disease
19	Lymphoma
20	Metastatic
21	Obesity
22	Other neurological
23	Paralysis
24	Peptic ulcer disease excluding bleeding
25	Peripheral
26	Psychoses
27	Pulmonary circulation disorders
28	Renal failure
29	Rheumatoid arthritis/collagen vascular disease
30	Solid tumor without metastasis
31	Valvular disease

AIDS refers to “acquired immunodeficiency syndrome”; HIV refers to “human immunodeficiency virus”.

The second specific regression model was not annual but aggregated per visit to the hospital. The model was as follows:


(2) $$\begin{array}{l} Y_{iclt}=\beta_{0}+\beta_{1}Treated_{iclt}+\beta_{2}Postpolicy_{iclt}+\beta_{3}Postpolicy_{iclt}\\ \;{}^{*}Treated_{iclt}+\beta_{4}Control_{iclt}+\varepsilon_{iclt} \end{array}$$

In this model, *i* in iclt denoted the patient ID, *c* denoted the prefecture-level city, *l* denoted the quarter of the visit or discharge time, and *t* denoted the type of visit (outpatient or inpatient). *Y*_*iclt*_ was the dependent variable with medical expenses and cost-sharing ratios. *Treated*_*iclt*_ denoted the dummy variables for the treatment and control groups, a value of 1 denoted the group with the change of health insurance reimbursement policies, 0 denoted the group without the change of health insurance reimbursement policies. *Postpolicy*_*iclt*_ indicated whether it was before or after the change of health insurance reimbursement policies, a value of 1 meant the time was after the policy change, while a value of 0 meant the time was before the policy change. $Postpolic{y_{iclt}}^{*}Treated_{iclt}$ was the interaction term that was of interest; the significance of its coefficient indicated whether the change of health insurance reimbursement policies in the treatment group had an impact on *Y*_*iclt*_ after considering the time effect of the control group, and if so, the direction of the impact. *Control*_*iclt*_ was a series of control variables, including patients' age and gender and 31 dummy variables for various comorbidities, which were described by Quan et al. (35) and can be found in Table 3. ε_*iclt*_ was the error term.

After illustrating the impact of policy change by DID models quantitatively. The event study method provided a qualitative trend visually. The event study was performed as follows:


(3) $$\begin{array}{l} Y_{iclt}=\lambda_{0}+\overset{\tau =8}{\underset{\tau =-m}{\sum }}{\beta_{\tau }}^{*}1\left(l-T_{c}=\tau \right)+\beta_{-m-1}\\ \;{}^{*}1\left(l-T_{c}\leq -m-1\right)+{\beta_{9}}^{*}1\left(l-T_{c}\geq 9\right)\\ \;+\lambda_{1}Control_{iclt}+\varepsilon_{iclt} \end{array}$$

In this model, *i* in iclt denoted the patient ID, *c* denoted the prefecture-level city, *l* denoted the quarter of the visit or discharge time, and *t* denoted the type of visit (outpatient or inpatient). *Y*_*iclt*_ were the dependent variables including medical expenses and cost-sharing ratios, as in model (2). *T*_*c*_ was the quarter of the policy change, 1(*l* − *T*_*c*_ = τ) denoted the dummy variable, a value of 1 meant that the time difference between the current quarter *l* and *T*_*c*_ was τ, otherwise a value of 0 was assigned; the model denoted the dummy variables from −*m* to 8 (−1 was treated as the control period and was, thus, not included) when *l* − *T*_*c*_ was less than –*m*, the dummy variable was uniformly set to ≤ -*m* – 1 and when *l* − *T*_*c*_ was >8, the dummy variable was uniformly set to ≥9. For example, the policy change in the city of Hangzhou was implemented on 1 January 2014, corresponding to the first quarter of 2014 (2014q1), therefore, if the hospital visit was 2014q2, its time distance from the policy change was 1. *Control*_*iclt*_ indicated control variables, such as age, gender, and 31 dummy variables for various comorbidities, which were described by Quan et al. (35) and can be found in Table 3; and time variables were added to control for time effects. ε_*iclt*_ was the error term.

As part of the robustness check, we analyzed data from 2014 to 2017 to assess the impact of the zero-markup policy implementation in the Zhejiang province, China on 1 April 2014 (36). The results remained robust even after excluding the months with potential data fluctuations due to the Spring festival. All data cleaning and analysis was performed using the statistical software STATA 17, and all the results reported in this study could be fully reproducible.

## 3. Results

### 3.1. Characteristics of study population

Six thousand seven hundred and sixty six patients and their corresponding electronic health insurance claim data were included in the baseline year 2013. Of these, 2,899 patients were from the four cities that underwent the policy change and were designated as the treatment group. Table 4 presented a comparison of the basic descriptive results between the treatment and control groups, both before and after the policy change.

**Table 4:** Characteristics of study population before and after policy change.

**Variables**	**Description**	**Before policy change**			**After policy change**		
		**Treatment (*****n*** = **2,899)**	**Control (*****n*** = **3,867)**	***P***	**Treatment (*****n*** = **2,833)**	**Control (*****n*** = **4,324)**	***P***
Age, years	≥ 60	68.8%	66.7%	0.066	69.0%	69.6%	0.567
Gender	Female	44.7%	42.0%	0.027	45.0%	41.3%	0.002
Charlson index	Mean (SD)	4.0 (2.3)	4.0 (2.4)	0.606	4.7 (2.4)	5.2 (2.4)	0.000
	0	6.1%	6.3%	0.656	3.0%	1.9%	0.002
	1–3	36.3%	37.1%	0.507	29.8%	24.0%	0.000
	≥ 4	57.6%	56.6%	0.388	67.3%	74.1%	0.000
**Medical expenses**							
Annual total expenses	Median (IQR)	4,470.3 (11,422.1)	4,476.1 (7,965.1)	0.980	10,080.1 (21,052.4)	7,981.3 (13,883.1)	0.000
Annual self-expenses within policy	Median (IQR)	1,096.6 (2,560.1)	1,229.5 (2,027.4)	0.037	2,072.9 (3,745.2)	2,114.6 (2,977.5)	0.618
Annual outpatient total expenses	Median (IQR)	3,116.0 (5,569.8)	3,732.7 (5,606.1)	0.000	6,605.5 (10,670.8)	5,809.3 (70,66.1)	0.000
Annual inpatient total expenses	Median (IQR)	14,048.2 (27,935.1)	10,991.3 (19,411.9)	0.001	19,117.3 (40,133.7)	14,514.0 (26,691.7)	0.000
**Cost-sharing ratios**							
Annual ratio of self-expenses within policy on total expenses	Mean (SD)	0.30 (0.30)	0.30 (0.24)	0.199	0.27 (0.29)	0.28 (0.21)	0.028
Annual outpatient ratio of self-expenses within policy on total expenses	Mean (SD)	0.31 (0.32)	0.32 (0.25)	0.364	0.28 (0.31)	0.31 (0.23)	0.000
Annual inpatient ratio of self-expenses within policy on total expenses	Mean (SD)	0.19 (0.10)	0.17 (0.10)	0.000	0.17 (0.11)	0.16 (0.12)	0.859
Annual inpatient out-of-pocket ratio	Mean (SD)	0.31 (0.14)	0.32 (0.11)	0.328	0.29 (0.13)	0.32 (0.12)	0.000
Annual inpatient copayment ratio within policy	Mean (SD)	0.22 (0.14)	0.19 (0.12)	0.000	0.19 (0.12)	0.19 (0.13)	0.515
**Health outcomes**							
Annual hospitalization rate	Rate	24.8%	18.2%	0.000	31.2%	32.1%	0.408
Rehospitalization rate within 30 days	Rate	14.3%	15.5%	0.525	25.6%	22.6%	0.097
Rehospitalization rate within 90 days	Rate	27.2%	23.3%	0.091	40.8%	38.4%	0.260

Wilcoxon test and t-test were used for the median and mean, respectively.

### 3.2. How the policy change affected medical expenses, cost-sharing ratios, and health outcomes

To examine the overall impact of the policy change on medical expenses, cost-sharing ratios, and health outcomes, we used the difference-in-differences (DID) model mentioned previously [model (1)]. The impact on medical expenses is presented in Table 5, which includes coefficients and a 95% confidence interval. Columns (1) and (2) report the estimate of annual medical expenses, including the annual total expenses, annual self-expenses within policy, and annual out-of-pocket expenses. Columns (3)–(5) report the estimate of annual inpatient medical expenses, and columns (6) and (7) report the estimate of annual outpatient medical expenses, respectively. The coefficients of Post policy^*^Treated are of particular interest. The results indicate that the policy change increased the annual outpatient total expenses by 29% (95% CI 24–34%) while decreasing the annual inpatient total expenses by 47% (95% CI 28–66%). After taking into account the offset between inpatient and outpatient expenses, we estimated that the policy change resulted in a 14% (95% CI 9–20%) increase in the annual total expenses.

**Table 5:** DID regression analysis of the effect of policy change on annual medical expenses.

	**(1)**	**(2)**	**(3)**	**(4)**	**(5)**	**(6)**	**(7)**
	**Annual total expenses**	**Annual self-expenses within policy**	**Annual inpatient total expenses**	**Annual inpatient self-expenses within policy**	**Annual inpatient out-of-pocket expenses**	**Annual outpatient total expenses**	**Annual outpatient self-expenses within policy**
Post policy	0.19^***^	0.15^***^	0.16	0.12	0.15	0.23^***^	0.22^***^
	(0.13, 0.24)	(0.07, 0.24)	(−0.05, 0.36)	(−0.04, 0.28)	(−0.03, 0.33)	(0.18, 0.28)	(0.14, 0.30)
Post policy^*^ treated	0.14^***^	0.034	−0.47^***^	−0.37^***^	−0.44^***^	0.29^***^	0.17^***^
	(0.09, 0.20)	(−0.05, 0.11)	(−0.66, −0.28)	(−0.53, −0.22)	(−0.61, −0.27)	(0.24, 0.34)	(0.09, 0.24)
Constant	8.03^***^	6.41^***^	5.57^**^	4.29^**^	4.92^**^	6.18^***^	4.80^***^
	(7.00, 9.06)	(4.83, 7.98)	(1.76, 9.38)	(1.26, 7.32)	(1.58, 8.26)	(5.24, 7.11)	(3.35, 6.24)
Observations	36,460	36,460	36,460	36,460	36,460	36,460	36,460

^***^p < 0.01, ^**^p < 0.05; The 95% confidence intervals for the corresponding regression coefficients are in parentheses. The regressions controlled for sex, age, and comorbidities with year fixed effects.

Table 6 shows the DID results for cost-sharing ratios. It indicates that after the policy change, the annual outpatient ratio of self-expenses within policy on total expenses, the annual inpatient ratio of self-expenses within policy on total expenses, and the copayment ratio within policy all decreased.

**Table 6:** DID regression analysis of the effect of policy change on annual cost-sharing ratios.

	**(1)**	**(2)**	**(3)**	**(4)**	**(5)**
	**Annual total**		**Inpatient**		**Outpatient**
	**Ratio of self-expenses within policy on total expenses**	**Ratio of self-expenses within policy on total expenses**	**Out-of-pocket ratio**	**Copayment ratio within policy**	**Ratio of self-expenses within policy on total expenses**
Post policy	−0.0076^*^	0.010^*^	−0.0015	0.0085	−0.0010
	(−0.01, −0.00)	(0.00, 0.02)	(−0.01, 0.01)	(−0.00, 0.02)	(−0.01, 0.00)
Post policy ^*^Treated	−0.0059	−0.021^***^	−0.012	−0.019^***^	−0.010^***^
	(−0.01, 0.00)	(−0.03, −0.01)	(−0.02, 0.00)	(−0.03, −0.01)	(−0.02, −0.00)
Constant	0.40^***^	0.23^***^	0.28^***^	0.24^***^	0.45^***^
	(0.28, 0.53)	(0.17, 0.28)	(0.21, 0.35)	(0.18, 0.30)	(0.34, 0.56)
Observations	36,460	10,203	10,203	10,196	36,286

^***^p < 0.01, ^*^p < 0.1; The 95% confidence intervals for the corresponding regression coefficients are in parentheses. The regressions controlled for sex, age, and comorbidities with year fixed effects.

Regarding health outcomes, as represented by the rehospitalization rate within 30 days and rehospitalization rate within 90 days, Table 7 shows that the policy change did not have a significant effect on the rehospitalization rate within 30 days but did lower the rehospitalization rate within 90 days.

**Table 7:** DID regression analysis of the effect of policy change on health outcomes.

	**(1)**	**(2)**	**(3)**
	**Hospitalization rate**	**Rehospitalized in 30 days**	**Rehospitalized in 90 days**
Post policy	0.17^*^	−0.0047	−0.057
	(0.01, 0.32)	(−0.37, 0.36)	(−0.38, 0.26)
Post policy ^*^treated	−0.42^***^	−0.0060	−0.39^*^
	(−0.57, −0.27)	(−0.40, 0.39)	(−0.72, −0.05)
Observations	20,909	3,888	4,880

^***^p < 0.01, ^*^p < 0.1; The 95% confidence intervals for the corresponding regression coefficients are in parentheses. The regressions controlled for sex, age, and comorbidities with year fixed effects.

To further examine how medical expenses per visit and cost-sharing ratios per visit responded to the policy change, we analyzed the results shown in Tables 8, 9. These results indicate that both inpatient and outpatient medical expenses per visit increased after the policy change.

**Table 8:** DID regression analysis of the effect of policy change on medical expenses per visit.

	**(1)**	**(2)**	**(3)**	**(4)**	**(5)**
	**Inpatient total expenses**	**Inpatient self-expenses within policy**	**Inpatient out-of-pocket expense**	**Outpatient total expenses**	**Outpatient self-expenses within policy**
Post policy	−0.12^***^	0.34^***^	−0.032	0.020^***^	−1.25^***^
	(−0.18, −0.06)	(0.24, 0.43)	(−0.10, 0.04)	(0.01, 0.03)	(−1.26, −1.24)
Treated	0.020	0.27^***^	−0.028	0.25^***^	−0.020^*^
	(−0.04, 0.08)	(0.19, 0.36)	(−0.09, 0.04)	(0.23, 0.26)	(−0.04, −0.00)
Post policy ^*^treated	0.24^***^	0.18^***^	0.093^*^	0.076^***^	0.077^***^
	(0.17, 0.31)	(0.08, 0.27)	(0.02, 0.17)	(0.06, 0.09)	(0.06, 0.10)
Constant	10.5^***^	8.65^***^	9.67^***^	4.42^***^	2.77^***^
	(9.43, 11.63)	(7.57, 9.74)	(8.35, 10.99)	(4.27, 4.57)	(2.53, 3.01)
Observations	19,926	19,926	19,926	1,024,640	1,024,640

^***^p < 0.01, ^*^p < 0.1; The 95% confidence intervals for the corresponding regression coefficients are in parentheses. The regressions controlled for sex, age, and comorbidities with year and month fixed effects.

**Table 9:** DID regression analysis of the effect of policy change on cost-sharing ratios per visit.

	**(1)**	**(2)**	**(3)**	**(4)**
	**Inpatient**			**Outpatient**
	**Ratio of self-expenses within policy on total expenses**	**Out-of-pocket ratio**	**Copayment ratio within policy**	**Ratio of self-expenses within policy on total expenses**
Post policy	0.039^***^	0.0070	0.033^***^	−0.23^***^
	(0.03, 0.05)	(−0.00, 0.02)	(0.03, 0.04)	(−0.24, −0.23)
Treated	0.012^***^	−0.0077	0.017^***^	0.0090^***^
	(0.00, 0.02)	(−0.02, 0.00)	(0.01, 0.03)	(0.01, 0.01)
Post policy ^*^treated	−0.020^***^	−0.028^***^	−0.014^***^	−0.028^***^
	(−0.03, −0.01)	(−0.04, −0.02)	(−0.02, −0.00)	(−0.03, −0.03)
Constant	0.18^***^	0.38^***^	0.19^***^	0.39^***^
	(0.17, 0.20)	(0.25, 0.51)	(0.17, 0.21)	(0.36, 0.43)
Observations	19,926	19,926	19,894	1,024,640

^***^p < 0.01; The 95% confidence intervals for the corresponding regression coefficients are in parentheses. The regressions controlled for sex, age, and comorbidities with year and month fixed effects.

**Figure 1:**
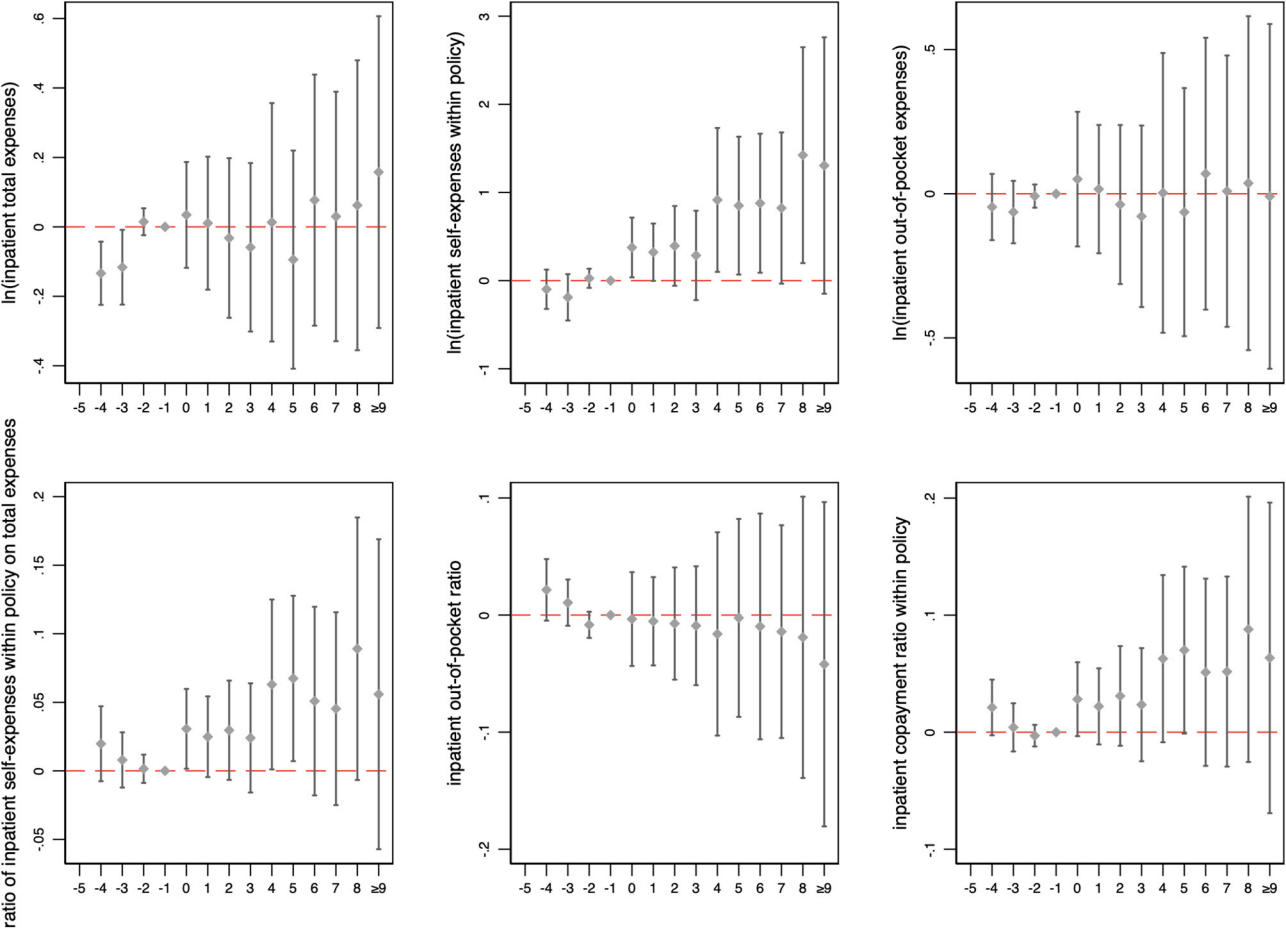
Changes in inpatient medical expenses and cost-sharing ratios over time. The figure illustrates the coefficient and 90% confidence intervals generated by event study. The horizontal axis represents the number of months relative to the month of policy change implementation.

**Figure 2:**
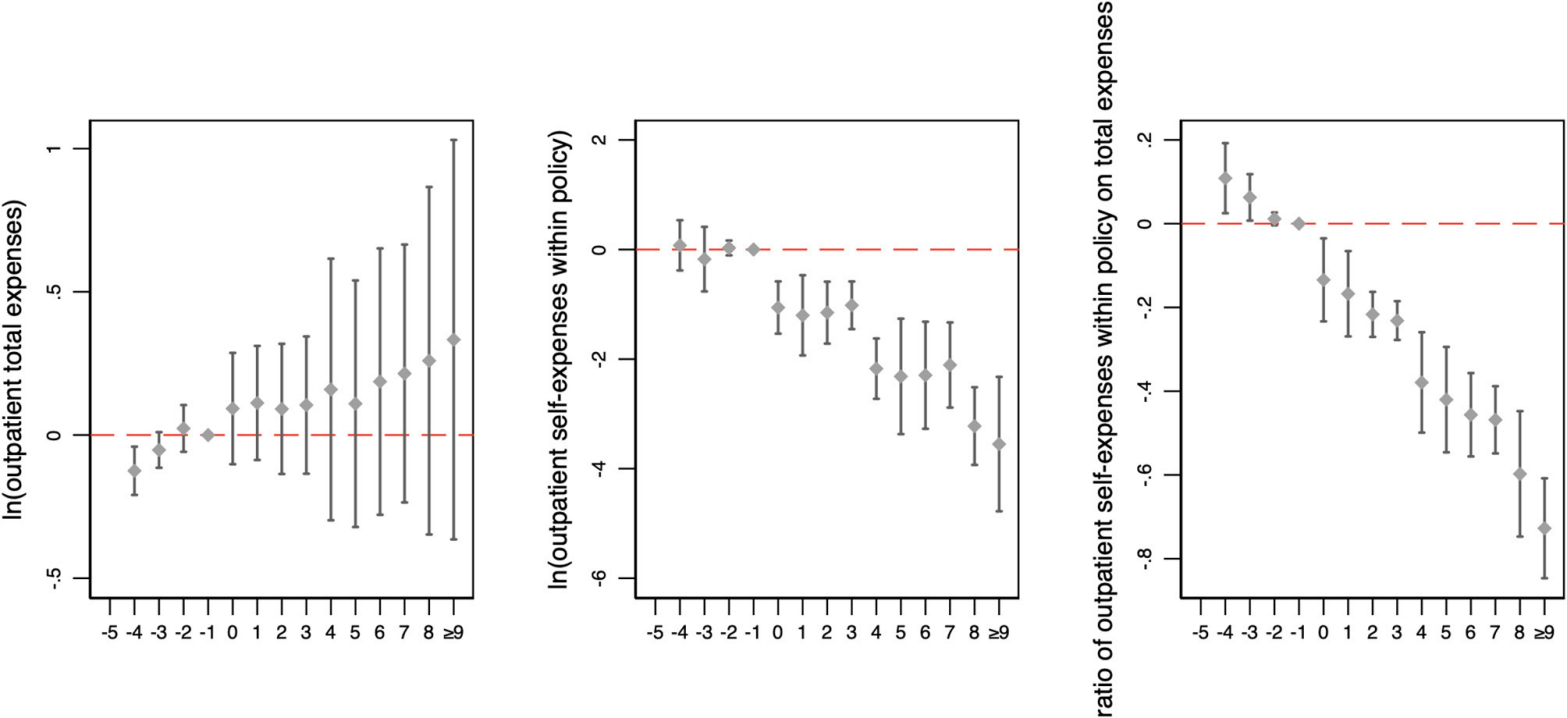
Changes in outpatient medical expenses and cost-sharing ratios over time. The figure illustrates the coefficient and 90% confidence intervals generated by event study. The horizontal axis represents the number of months relative to the month of policy change implementation.

The results of the event study are presented in Figures 1, 2. The coefficients of inpatient medical expenses and cost-sharing ratios were almost all insignificant compared to the quarter before the policy change. Figure 2 shows that for outpatient expenses and cost-sharing ratios, the coefficients of outpatient total expenses were also insignificant in all periods, while the outpatient cost-sharing ratios continued to decrease after the policy change.

## 4. Discussion

This study evaluates the impact of changes to the UEBMI reimbursement policies aimed at reducing patient cost-sharing on medical expenses and health outcomes among patients with heart failure. The findings indicate limited impact. While the total medical expenses per visit increased for both outpatient and inpatient care in response to lower patient cost-sharing (Tables 8, 9), as previously reported in the literature (7, 14), our results show that the annual total expenses in the treatment group did not decrease compared to the control group (Table 5). This suggests that the policy change did not result in a reduction in the economic burden faced by patients with heart failure from a societal perspective. With regard to health outcomes, this study has found that the policy change was associated with a reduced likelihood of rehospitalization within 90 days, but not within 30 days (Table 7), which aligned with the findings of a study in the acute coronary syndrome population (37). One possible mechanism for reducing medical expenses through lower patient cost-sharing is that patients can choose higher-quality therapies to delay disease progression and reduce hospitalization rates, thereby reducing annual medical expenses. However, this study shows that the reduction in patient cost-sharing did not reduce annual medical expenses, which may be explained by the fact that the reduction in patient cost-sharing did not effectively increase the availability of higher-quality therapies. Additionally, despite the reduced patient cost-sharing, many cost-effective drugs or therapies were not included in the medical insurance catalog.

Patient cost-sharing plays an important role in influencing physician and patient behavior, directly and subsequently affecting the reallocation of medical services. This study shows that the impact of the patient cost-sharing on medical expenses differed between outpatients and inpatients. On a micro level, lower patient cost-sharing for outpatients resulted in higher annual medical expenses and expenses per visit (Tables 5, 8). This increase in annual outpatient total expenses led to an overall increase in the annual total expenses, even though annual inpatient total expenses decreased (Table 5). Therefore, from a societal perspective, the policy change did not reduce the medical burden on patients with heart failure. This finding indicates that the reimbursement policies may focus on how to reduce the patient cost-sharing of outpatients in the future. On a macro level, patient cost-sharing can directly impact the economic burden of patients, which can then influence the proportion of the disease burden among all illnesses. Policymakers may use figures on the proportion of the disease burden to inform policy-making decisions, which can, in turn, indirectly impact the economic burden for patients with heart failure. In our study, even though the policy change decreased the inpatient copayment ratio within policy among inpatients, both per visit and annually, it had no effect on the inpatient out-of-pocket ratio or annual total ratio of self-expenses within policy on total expenses (Table 6), which were more concerning to patients. As a result, the findings of this study have significant implications for practical applications. Policymakers may need to consider incorporating reimbursement policies into other medical insurance policies, for example by including more cost-efficient drugs or services in the medical insurance reimbursement catalog. These policies should take into account the unique characteristics of the disease, such as treating heart failure as a special outpatient disease and increasing the reimbursement ratio for outpatient care. These measures would effectively reduce the economic burden on patients with heart failure and improve their overall health outcomes.

The limitations of this study should be considered when interpreting the results. First, the study only focused on a limited number of health outcomes and did not consider the impact of patient cost-sharing on other important health outcomes, such as mortality, due to the unavailability of data. Nevertheless, the hospitalization and rehospitalization rates are widely used and are representative indicators. Second, the study was unable to separate out-of-pocket expenses for outpatients due to a lack of information on outpatient insurance expenses within policy. Third, the study did not control for important patient demographics, such as income and education level, due to a lack of data from other sources. To better understand the impact of patient cost-sharing on medical expenses and health outcomes, future studies should employ multiple sources of data, consider a broader range of health outcomes, and incorporate relevant patient demographics.

## 5. Conclusion

Our study found that the impact of the policy change on medical expenses and health outcomes was modest. While the patient cost-sharing ratio (specifically the copayment ratio within policy) decreased, the annual total expenses for heart failure patients did not. This indicates that further efforts, such as expanding the health insurance reimbursement catalog to cover more drugs and items, may be needed to relieve patients of medical expenses. These results emphasize the need for ongoing efforts to lower cost-sharing ratios, particularly the out-of-pocket ratio for patients.

## Data availability statement

The data analyzed in this study is subject to the following licenses/restrictions: the data used in this study are nonpublic electronic Urban Employees' Basic Medical Insurance claim records belonging to Shanghai Songsheng Business Consulting Co. Ltd. Requests to access these datasets should be directed to JW, bonejizi@126.com.

## Ethics statement

Ethical review and approval was not required for the study on human participants in accordance with the local legislation and institutional requirements. Written informed consent for participation was not required for this study in accordance with the national legislation and the institutional requirements.

## Author contributions

HZ designed the study under the supervision of HF. JW contributed to data collection and discussion. HZ carried out the data analysis and wrote the manuscript. HZ, KN, and HF contributed to the interpretation of the results and revised the manuscript. All authors have read and agreed to the published version of the manuscript.
